# Position-Dependent Effects of AP Sites Within an *hTERT* Promoter G-Quadruplex Scaffold on Quadruplex Stability and Repair Activity of the APE1 Enzyme

**DOI:** 10.3390/ijms26010337

**Published:** 2025-01-02

**Authors:** Viktoriia Yu. Savitskaya, Kirill A. Novoselov, Nina G. Dolinnaya, Mayya V. Monakhova, Viktoriia G. Snyga, Evgeniia A. Diatlova, Elizaveta S. Peskovatskova, Victor M. Golyshev, Mariia I. Kitaeva, Daria A. Eroshenko, Maria I. Zvereva, Dmitry O. Zharkov, Elena A. Kubareva

**Affiliations:** 1 Department of Chemistry, Lomonosov Moscow State University, 119991 Moscow, Russia; svk1896@mail.ru (V.Y.S.); dolinnaya@hotmail.com (N.G.D.); snyga.viktoria000@gmail.com (V.G.S.); makita01@mail.ru (M.I.K.); maria.i.zvereva@yandex.ru (M.I.Z.); 2 A.N. Belozersky Institute of Physico-Chemical Biology, Lomonosov Moscow State University, 119991 Moscow, Russia; monakhovamv@gmail.com; 3 Institute of Chemical Biology and Fundamental Medicine, Siberian Branch of the Russian Academy of Sciences, 630090 Novosibirsk, Russia; diatlova@niboch.nsc.ru (E.A.D.); golyshevvictor@gmail.com (V.M.G.); daraero19@gmail.com (D.A.E.); dzharkov@niboch.nsc.ru (D.O.Z.); 4 Department of Bioengineering and Bioinformatics, Lomonosov Moscow State University, 119234 Moscow, Russia; elizavetapeskovatskova@gmail.com; 5 Department of Natural Sciences, Novosibirsk State University, 630090 Novosibirsk, Russia

**Keywords:** apurinic/apyrimidinic site, G-quadruplex, DNA repair, base excision repair, APE1, *hTERT* promoter, gene expression

## Abstract

Apurinic/apyrimidinic (AP) sites are endogenous DNA lesions widespread in human cells. Having no nucleobases, they are noncoding and promutagenic. AP site repair is generally initiated through strand incision by AP endonuclease 1 (APE1). Although AP sites’ repair in regular B-DNA has been studied extensively, their processing in G-quadruplexes (G4s) has received much less attention. Here, we used the *hTERT* promoter region that is capable of forming three stacked parallel G4s to understand how AP sites can influence higher-order quadruplex folding and stability and how a G4 affects the efficiency of human APE1-mediated AP site processing. We designed a series of synthetic single- and double-stranded DNA constructs of varying lengths containing a stable AP site analog in both G- and C-rich strands at positions corresponding to somatic driver mutations. Using circular dichroism, we studied the effect of the AP site on *hTERT* G4 structure and stability. Bio-layer interferometry and gel-based approaches were employed to characterize APE1 binding to the designed DNA substrates and AP site processing. It was shown that (i) an AP site leads to G4 destabilization, which depends on the lesion location in the G4 scaffold; (ii) APE1 binds tightly to *hTERT* G4 structure but exhibits greatly reduced cleavage activity at AP sites embedded in the quadruplex; and (iii) a clear correlation was revealed between AP site-induced *hTERT* G4 destabilization and APE1 activity. We can hypothesize that reduced repair of AP sites in the *hTERT* G4 is one of the reasons for the high mutation rate in this promoter region.

## 1. Introduction

Apurinic/apyrimidinic (AP or abasic) sites are some of the most common types of DNA lesions found in human cells [1]. AP sites arise from cleavage of the *N*-glycosidic bond between a nucleobase and the deoxyribose moiety, leaving the phosphodiester backbone intact. The base loss can occur either spontaneously or as a result of cell exposure to X-ray or UV irradiation or various chemicals. AP sites are also generated through DNA glycosylase–catalyzed removal of damaged bases in the first stage of base excision repair (BER) [2,3,4,5,6]. If an AP site is not repaired prior to the passage of a replication fork, then such a site can block DNA polymerases, in particular α, δ, and ε [6,7], and thus can compromise replication. If these noncoding lesions are bypassed by polymerases during DNA synthesis, then they serve as a potent source of mutations. In addition, AP sites, due to their reactivity, can be converted to lesions more challenging for repair, including double-strand breaks, interstrand cross-links [8,9,10], and DNA–protein cross-links [11]. Therefore, understanding the AP site repair mechanisms that prevent the accumulation of mutations and ultimately carcinogenesis [12] is important.

Given the prevalence of AP sites and their deleterious consequences, cells possess multiple mechanisms to repair or tolerate these lesions. In mammalian cells, the repair of AP sites is generally initiated through strand incision by AP endonuclease 1 (APE1): an enzyme in the canonical BER pathway [13]. The evolutionarily conserved BER system operates in both nuclei and mitochondria, thereby ensuring the maintenance of genomic stability [2]. In human cells, multifunctional APE1 specifically recognizes AP site-induced local conformational changes of a DNA double helix and cleaves the sugar phosphate backbone 5′ to an AP site to produce a single-nucleotide gap flanked by a 2′-deoxyribose 5′-phosphate and a 3′-hydroxyl group [14]. In addition, this enzyme is engaged in transcriptional regulation [15,16,17]. High-resolution crystal structure of human APE1 in complex with double-stranded (ds) DNA containing a centrally located stable AP site analog (1,2-dideoxyribofuranose, F-site) has revealed key aspects of the reaction mechanism: APE1 flips the AP site out of the double helix into the active site’s binding pocket, leaving an orphaned base in the opposite strand [18,19,20]. It has been established that amino acid substitutions in the active site of APE1 lead to APE1 activity deficiencies [21] associated with an increase in the frequency of spontaneous mutations, with increased susceptibility to cancer, and with reduced survival after exposure to exogenous oxidizing agents [22,23,24,25,26].

The nuclease function of APE1 is well characterized on dsDNA substrates, where the complementary strand provides a template for subsequent BER events. In addition, this enzyme possesses moderate AP endonuclease activity on single-stranded (ss) DNA regions, including those in various biologically relevant DNA structures such as bubbles or R-loops [18,27,28]. Of note, APE1 is only weakly active on AP site–containing ss polyA and ss polyT sequences [29], suggesting that some DNA secondary structure is required for efficient cleavage. The tight packaging of eukaryotic cellular DNA into chromatin also limits APE1’s access to AP sites.

New interest in AP site biology arose from recent insights into how they are processed in noncanonical G-quadruplex (G4) DNA [15,30,31]. Endogenous G4s form through a conformational transition of ssDNA sequences containing four or more repeating G-tracts. They fold intramolecularly to form planar, stacked G-tetrads stabilized by Hoogsteen hydrogen bonds between adjacent guanine residues. The G4 core is further stabilized by the coordination of monovalent cations, usually potassium ions, with guanine O6 atoms. Depending on the length and composition of G4-forming sequences (G4 motifs), G4s can vary in stability and adopt various topologies, including parallel, antiparallel, or hybrid (3 + 1), which differ in the orientation of the G-tracts [32]. Computational analysis has predicted that nearly one million unique G4 motifs in the human genome are capable of folding into endogenous G4s [33]. In addition, there is strong evidence that G4s can form dynamically within live cells [34]. For instance, approximately 10,000 G4 structures have been shown to exist at any given moment in a human cell [35]. Genomic G4s are involved in the regulation of various physiological processes, e.g., telomere maintenance, DNA replication, transcription, repair, and cell differentiation [36,37,38,39,40,41]. On the other hand, G4s may also contribute to genomic instability by serving as physical obstacles to DNA polymerase processivity and by promoting DNA damage and recombination events that are linked to tumorigenesis and other genetic disorders. Recent studies emphasize connections between genomic instability, DNA repair activity, and quadruplex formation [42]. These G4 structures effectively bind to various cellular proteins participating in DNA repair [38], thereby affecting their primary functions.

So far, the interaction of APE1 with quadruplex DNA has been studied in a limited set of models, mainly G4s from promoter regions of human genes *VEGF*, *KRAS*, and *NEIL3* and telomeric repeats. APE1 has been found to poorly cleave the DNA backbone at an abasic lesion in the promoter G4s but to bind tightly to it, although it remains unclear whether this binding directly involves the AP site [31,43,44,45]. In telomeric G4s, cleavage by APE1 is more favorable but is highly dependent on the nature and concentration of mono- and divalent cations as well as on the position of the lesion within the G4 structure [15,46,47]. The binding appears to be modulated by post-translational modification of several Lys and Cys residues in the protein [15,31,47]. In the context of cellular chromatin, APE1 binding to potential quadruplex-forming sequences appears to promote G4 folding [12,48].

**Figure 1:**
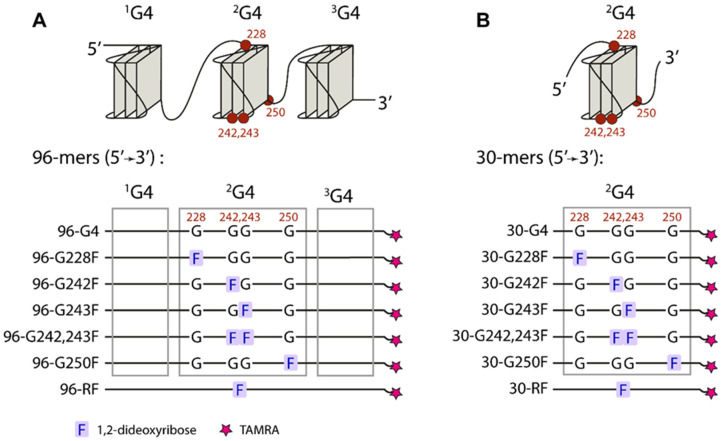
Schematic representation of synthetic ssDNAs identical to the *hTERT* promoter G-rich strand sequence. Folded G4 structures and positions of G>F point substitutions (corresponding to driver mutations), marked by red circles, are shown in the top panel. The G4 motifs forming the three hTERT quadruplex units (^1^G4, ^2^G4, and ^3^G4) are indicated. (**A**) The unmodified 96-nt construct (96-G4) and its variants with substitution G228F, G242F, G243F, G242,243F, or G250F (designated as 96-G228F, 96-G242F, 96-243F, 96-G242,243F, and 96-250F, respectively). (**B**) The set of 30-nt constructs, including those harboring G>F substitutions at positions similar to those in 96-nt DNAs (30-G4 unmodified oligonucleotide, 30-G228F, 30-G242F, 30-243F, 30-G242,243F, and 30-250F). The 96- and 30-nt control DNAs, which contain mid-strand F residues but are unable to form G4 structures, are designated as 96-RF and 30-RF, respectively. Pink asterisks denote the TAMRA fluorophore attached to the 3′ end of the oligonucleotides.

To better understand the cancer progression mechanism, noncoding regions of the genome, where mutations occur at a very high frequency, are being actively investigated. One key region of interest is the G-rich promoter sequence of the human telomerase reverse transcriptase (*hTERT*) gene. This gene encodes the hTERT enzyme, which is crucial for telomere maintenance and immortality of cancer cells [49]. In most somatic cells (except stem cells), hTERT is inactive, but its abnormal expression is associated with 85–90% of cancers [49]. Recent genetic studies revealed several driver mutations in the core *hTERT* promoter, particularly in the region approximately spanning positions −180 to +1 from the transcription start site. The most common mutations result from C>T substitutions in the C-rich coding strand of the *hTERT* gene. They occur at positions −124 and −146 (i.e., 1,295,228 and 1,295,250 on chromosome 5) relative to the *hTERT* gene start codon ATG and are designated as C228T and C250T, respectively. These mutations are detectable in 60–80% of urothelial carcinomas [50], 71% of melanomas [51], 83% of glioblastomas [52], and many other types of cancer. Rarer double substitution CC>TT at positions −138 and −139 (C242T and C243T) occurs predominantly in skin tumors. These mutations create new binding sites, 5′-CCGGAA-3′/5′-TTCCGG-3′, for the ETS (E-twenty-six) transcription factor family and provide a selective advantage to liver, glioblastoma, and bladder cancer cells by recruiting a multimeric GA-binding protein (GABP) transcription factor specifically to the mutant *hTERT* promoter. In the G-rich template strand of the *hTERT* gene, these clinically relevant mutations arise from G>A substitutions (G250A, G228A, G242A, and G243A); they are located in a 68-nucleotide (nt) G-rich region [53,54]. This region contains 12 G-tracts of three or more consecutive guanines, and these tracts are capable of forming multiquadruplex structures. In our previous work, we used spectroscopic and chemical probing methods to demonstrate that 96-nt ssDNAs, which model the G-rich strand of both the wild-type (WT) *hTERT* promoter and its mutated versions, fold into three stacked parallel G4s [55]. The guanines most susceptible to mutation fall into G-tracts 5, 7, and 8, which give rise to the central G4 unit in this three-quadruplex structure of the *hTERT* promoter (Figure 1A).

Low redox potential of guanine often makes G-rich sequences susceptible to oxidizers and prone to the emergence of AP sites after base excision or spontaneous base loss [56,57]. Such damage in G4-forming sequences may affect G4 stability and reduce AP site processing by the BER system, thus ultimately leading to the accumulation of mutations [57,58]. Notably, the APE1 endonuclease poorly cleaves AP sites located in human telomere G4s [31,47] or in parallel G4s arising in promoter regions of *c-MYC* [30] and *VEGF* [15]. Nonetheless, no data have been reported on APE1 activity on AP site–bearing multimeric *hTERT* quadruplex structures.

In this work, we deal with the impact of a higher-order *hTERT* G4 structure formed within 96-nt synthetic constructs on the efficiency of human APE1-mediated processing of AP sites modeled by F residues located at positions corresponding to driver mutations (Figure 1A). Shorter G- or F-containing 30-nt fragments of the G-rich *hTERT* promoter strand encompassing mutation sites were also employed as model systems (Figure 1B). Using circular dichroism (CD) spectroscopy, gel-based assays, and bio-layer interferometry, we elucidated how the position of the G>F substitution in the *hTERT* G4 motif can influence quadruplex stability, APE1 binding affinity, and catalytic activity. Because AP lesions in the C-rich *hTERT* promoter strand destabilize dsDNA and can trigger refolding of the complementary G-rich strand into the G4 structure, we also evaluated the positional effect of C>F substitutions on the efficiency of APE1-induced cleavage of AP sites. The findings provide new evidence supporting our hypothesis that the high mutation frequency in G/C-rich oncogene promoters is associated with G4-driven attenuation of DNA repair capacity; this phenomenon may play an essential role in some gene expression steps.

## 2. Results

### 2.1. Bioinformatic Analysis of the Simultaneous Occurrence of Mutations in the APEX1 Gene and the hTERT Promoter

In human cells, APE1 initiates repair of AP sites (an abundant type of DNA lesion), whereas substitutions of critical amino acid residues in the enzyme can destabilize the genome and contribute to tumor initiation and progression [26]. As described above, G>A substitutions in the G-rich strand of the *hTERT* promoter that are often located at positions G228, G242, G243, and G250 in multiple types of cancer create binding sites for ETS family transcription factors that increase the efficiency of *hTERT* gene transcription [50,51,52,54]. The reason for the high mutation frequency in this region is unclear, but the *hTERT* promoter strand corresponding to the template strand of the gene is G-rich and therefore prone to oxidative damage resulting in AP sites [44,57]. Inactivation of APE1 as a consequence of amino acid substitutions may be one of the reasons for the ineffective repair of AP sites in the *hTERT* promoter, and this inefficiency leads to the fixation of point mutations and activation of telomerase.

To assess the correlation between the presence of mutations in the *hTERT* promoter and in the *APEX1* gene, we queried the ICGC database. The G>A substitution at position G228 of *hTERT* was present in almost all 15 cancer types examined. The occurrence of mutation at position G250 was more common than at position G228 in the genomes of patients with melanoma and skin adenocarcinoma. G>A substitutions at positions G242 and G243 were present only in genomes of melanoma patients.

Among patients with mutations at the analyzed positions of the *hTERT* promoter, *APEX1* gene mutations were found only in melanoma patients from Australia. Of these, 1.6% had a mutation at position G250 of *hTERT* and less than 1% at position G228, G242, or G243 (Table 1).

Nevertheless, there was no statistically significant difference between the number of Australian melanoma patients with simultaneously identified mutations in *hTERT* and *APEX1* and the number of patients in whose genome there were no coincident mutations in *hTERT* and *APEX1*. Fisher’s exact test odds ratio and *p*-value for four kinds of simultaneous mutations were (1.6, 1.0), (1.6, 1.0), (1.6, 1.0), and (2.5, 0.6). Thus, we did not see a tendency for the simultaneous appearance of mutations at the analyzed positions of the *hTERT* promoter and the *APEX1* gene, and the high frequency of mutations in the *hTERT* promoter sequence is apparently not the result of APEX1 inactivation. This observation prompted us to investigate whether the quadruplex structure hampers the repair of AP sites in the *hTERT* G4s by fully functional APE1.

### 2.2. Design of DNA Models

To monitor APE1 action on *hTERT* G4s, we used a synthetic ss 3′-TAMRA-labeled 96-nt *hTERT* promoter fragment with a conserved 68-nt G4 motif bearing 12 G-tracts flanked by 14-nt sequences or its modified variants with G>F substitutions within the G4 motif: G228F, G242F, G243F, G250F, and double substitution G242,243F (Figure 1A; Supplementary Table S1, see Section 2.2), corresponding to somatic driver mutations. Folded structures with three stacked parallel G4s and G>F substitutions are shown in the top panel of Figure 1A. Taking into account that in the overall *hTERT* multiquadruplex structure (Figure 1A), all DNA lesions are located in G-tracts 5, 7, and 8 (falling into the central G4), we also constructed shortened 3′-TAMRA-labeled 30-nt variants of the *hTERT* G4 motif containing G>F substitutions at the same positions as in the 96-nt constructs (Figure 1B). These truncated DNA models make it possible to explain the influence of flanking unmodified G4s on F-mediated effects on quadruplex structure and thermal stability as well as on APE1 repair activity (Supplementary Table S1, Figure S1B). In particular, in 30-nt models, it is not possible to replace the AP site-carrying G-tract with another one located next to the damaged G4 [59]. As controls, we chose 96- and 30-nt DNA models containing intact *hTERT* G4 motifs (96-G4 and 30-G4, respectively) as well as 96- and 30-nt DNAs harboring F residues in the middle of the strand but unable to form G4 structures (96-RF and 30-RF, respectively) (Supplementary Table S1). For comparison, we used 96-bp DNA duplexes of the same sequence containing G>F substitutions in the G-rich strand (opposite C) or C>F substitutions in the C-rich strand (opposite G; Supplementary Table S2). DNA duplexes lacking G4 motifs served as controls (Supplementary Table S2).

### 2.3. Position-Dependent Effects of AP Sites on the Topology and Thermal Stability of hTERT G4s

**Figure 2:**
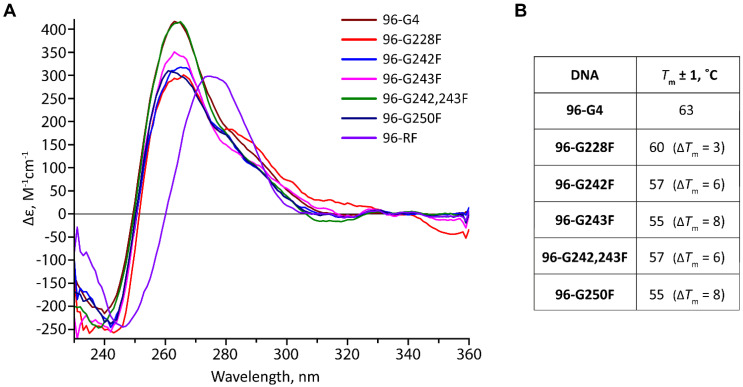
The position-dependent influence of AP (F) sites on the topology and thermal stability of the *hTERT* G4s. (**A**) CD spectra of 96-nt G-rich *hTERT* promoter strands bearing G>F substitutions (Figure 1) were recorded at 30 °C in 8 mM potassium phosphate buffer (pH 7.1) with 20 mM KCl. (**B**) *T*_m_ values obtained from CD-monitored thermal denaturation profiles of 96-nt *hTERT* G4s at 265 nm (Supplementary Figure S1) are presented along with the G4-destabilizing effects (Δ*T*_m_) induced by G>F substitutions as compared to unmodified construct 96-G4.

The topology and thermal stability of G4 structures arising in 96-nt constructs featuring various positions of G>F substitutions were analyzed by CD spectroscopy in 8 mM potassium phosphate buffer (pH 7.1) containing 20 mM KCl, which was commonly used to study *hTERT* G4 folding and thermodynamics [55]. WT 96-G4 yielded a CD spectrum with a maximum around 265 nm and a minimum near 240 nm, which are generally indicative of a G4 fold with all parallel G-tracts (Figure 2A). The CD spectrum of the 96-nt construct lacking the G4 motif (96-RF) but carrying an F residue in the middle of the molecule showed a positive peak at 280 nm, indicating the absence of a G4 structure. CD measurements of modified analogs of 96-nt G4 suggested that G>F substitutions within the quadruplex scaffold promote the formation of an antiparallel (hybrid) G4 topology manifesting itself in a positive shoulder at >280 nm; the only exception was 96-G242,243F, which clearly had a parallel topology. Unstructured oligonucleotides that flank the multiquadruplex also contribute to a shoulder at >280 nm. Nevertheless, the parallel G-tract direction still predominated in the AP site–containing quadruplex structure. From CD spectra recorded at different temperatures (Supplementary Figure S1), melting profiles of unmodified construct 96-G4 and its G>F substitution–bearing analogs were derived. The *T*_m_ values and F-induced quadruplex-destabilizing effects (Δ*T*_m_) are depicted in Figure 2B. CD-monitored melting curves reflect the superposition of the unfolding of three stacked quadruplex units, ^1^G4, ^2^G4, and ^3^G4 (Figure 1), which are stabilized by an equal number of G-tetrads [55]. Although the structure-destabilizing AP sites (F) were located only in the central ^2^G4, we observed position-dependent changes in both the cooperativity of G4 unfolding and the magnitude of the damage-induced G4 destabilizing effect (Supplementary Figures S1 and S2B), which ranged from 3 to 8 °C. G4 destabilization was less pronounced with the G228F substitution, likely owing to possible remodeling of the G4 fold by recruitment of adjacent dG from a long G-tract containing five consecutive G residues.

**Figure 3:**
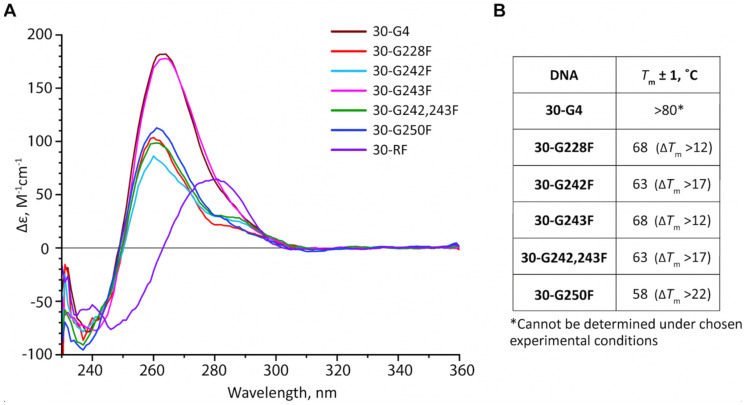
The position-dependent influence of AP (F) sites on the folding topology and thermal stability of the *hTERT* central ^2^G4 unit. (**A**) CD spectra of 30-nt G-rich *hTERT* promoter strands bearing G>F substitutions (Figure 1) were recorded at 25 °C in 10 mM Tris-HCl buffer (pH 8.0) supplemented with 1 mM EDTA and 100 mM KCl. (**B**) *T*_m_ values calculated from CD-monitored thermal denaturation profiles of 30-nt hTERT ^2^G4 at 265 nm (Supplementary Figure S2) are presented along with the G4-destabilizing effects (Δ*T*_m_) induced by G>F substitutions compared to unmodified 30-G4.

Nonetheless, it cannot be ruled out that the modified central ^2^G4 does not form at all, at least for 96-G250F harboring a G>F substitution in the shortest 3-nt G-tract, which is incapable of remodeling and causes maximum destabilization (8 °C) of the quadruplex ensemble. In principle, the observed CD spectra and melting profiles could be due mostly or entirely to the flanking unmodified *hTERT* G4 units. Therefore, to eliminate the effect of flanking quadruplex units, instead of the full-length 96-nt *hTERT* constructs, we next tested truncated 30-nt variants corresponding to the central ^2^G4, which carries all driver mutations. CD measurements were performed under experimental conditions that mimic those of APE1-induced DNA cleavage experiments (10 mM Tris-HCl buffer (pH 8.0) containing 1 mM EDTA and 100 mM KCl). The change in pH from 7.1 to 8.0 was solely due to optimization of the enzyme’s operation conditions. It was shown that the stability and topology of G4s do not change in this pH range [60]. The CD spectrum of the 30-nt construct lacking the G4 motif (30-RF) but carrying an F residue in the middle of the molecule shows a positive peak at 280 nm, indicating the absence of G4 structure (Figure 3A). In contrast, the spectral features of 30-G4 and 30-G243F clearly indicate the folding of G4 structures with a parallel topology. All other F-containing 30-nt variants of the *hTERT* central ^2^G4 unit yielded positive shoulders at wavelengths > 280 nm, indicating a subpopulation of antiparallel-folded G4s and a contribution of unstructured oligonucleotides flanking the central ^2^G4 (Figure 3A).

The CD-monitored melting profiles obtained from CD spectra recorded at different temperatures (Supplementary Figure S2) showed that the 30-nt constructs gave rise to significantly stabler G4 structures compared to the 96-nt ones owing to the 5-fold higher KCl concentration in the buffer; thus, unfolding of unmodified 30-G4 was incomplete even at 80 °C. On the other hand, the F-substituted 30-G4 analogs were clearly much less thermally stable, with Δ*T*_m_ values ranging from >12 to >22 °C (Figure 3B). As one can see in the CD results, the extent of G>F-induced quadruplex destabilization and topology changes depended on the position of the abasic site within the G4 scaffold. A guanine substitution in the shortest 3-nt G-tract (30-G250F) exerted the greatest destabilizing effect (Δ*T*_m_ > 22 °C) because no guanine adjacent to the lesion could be recruited for quadruplex folding. The G4 destabilization induced by a single G>F substitution at the first (or last) position of 4- or 5-nt G-tracts (30-G243F and 30-G228F) was significantly less (Δ*T*_m_ >12 °C) because these damaged G-tracts retain at least three consecutive Gs to form a quadruplex. On the other hand, a G>F substitution in the middle of a 4-nt G-tract (30-G242F) rendered only two consecutive guanines available for G-tetrad formation, thereby destabilizing this G4 more markedly (Δ*T*_m_ > 17 °C). Thus, single G>F substitutions in G-tracts forming the central ^2^G4 unit destabilize to some extent but do not completely disrupt the quadruplex structure.

### 2.4. APE1-Mediated Cleavage of AP (F) Sites Located in the G4 Scaffold Formed by the G-Rich Strand of the hTERT Promoter, as Well as AP Sites Locate in the Respective DNA Duplexes

**Figure 4:**
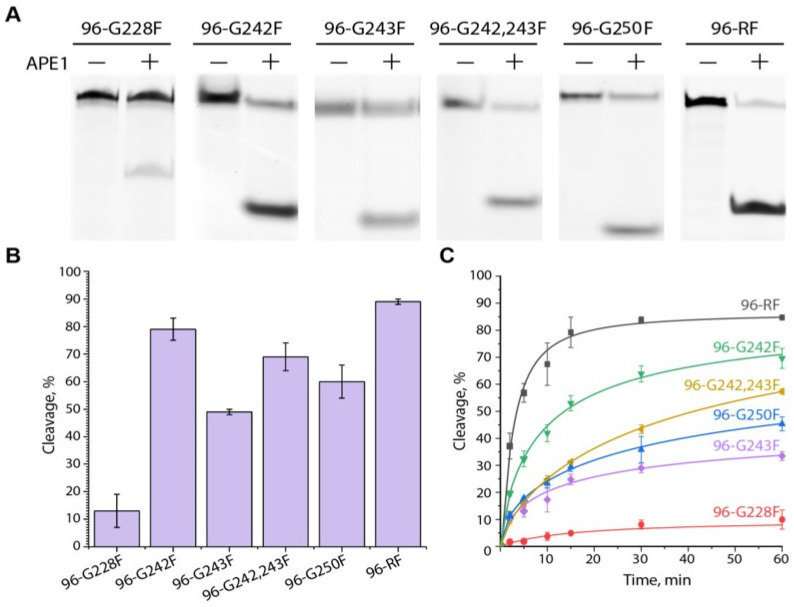
APE1-catalyzed F site cleavage in 96-nt *hTERT* G4 constructs. Reaction mixtures were composed of 20 mM Tris-HCl (pH 7.5), 100 mM KCl, 5 mM MgCl_2_, 1 mM DTT, 0.5 mg/mL BSA, 100 nM 3′-TAMRA-labeled ssDNA substrate, and 10 U (36 nM) APE1. The reactions were allowed to proceed at 37 °C for 60 min and quenched by heating at 60 °C for 20 min. (**A**) Representative gel images of 3′-TAMRA-labeled cleavage products after the 60-min reaction. (**B**) Cleavage efficiency as a function of the F site’s position as determined from the PAGE data. (**C**) Time courses of APE1-catalyzed DNA cleavage. Error bars are 95% confidence intervals (n = 3).

Wild-type recombinant APE1 was isolated as described [61]. The effect of the higher-order *hTERT* G4 structure on the APE1 endonuclease activity was investigated in a buffer containing 100 mM KCl, which favors G4 formation and is close to the concentration in the cell (nuclear K^+^ concentration is even higher and often exceeds 200 mM) [62,63]. The 96-nt *hTERT* multiquadruplex constructs bearing G>F substitutions in the central ^2^G4 core served as the substrates. Although the primary substrate of APE1 is dsDNA carrying an abasic site, the enzyme also acts on ssDNA but with lower efficiency [18,27,28]; here we first compared enzymatic activity on the F-containing ssDNA that is capable of forming G4s and on unstructured ssDNA substrates. APE1 cleaved DNA at F sites, thereby generating shorter products that were separated by polyacrylamide gel electrophoresis (PAGE) under denaturing conditions. All these DNAs were 3′-labeled with TAMRA to visualize and quantify bands (Figure 4A,B). APE1 cleavage in the G4 context was greatly attenuated as compared with that in control 96-RF lacking the G4 structure. The final yield of APE1-catalyzed cleavage products (after a 60-min reaction) was highly dependent on the F site location in the G4 scaffold and whether it was positioned in a 3-, 4-, or 5-nt G-tract. APE1-catalyzed cleavage activity tended to increase when thermal stability of the F-containing *hTERT* G4 structure decreased. For instance, a G>F substitution in a 5-nt G-tract caused the least destabilization of the quadruplex fold in 96-G228F, which was the worst APE1 substrate (final yield of the cleaved product ∼12%) (Figure 4B). This trend persisted over the time range from 2 to 60 min (Figure 4C).

Hybridization of the promoter G4 motifs to fully complementary DNA strands is known to dramatically diminish the propensity for G4 formation, even under conditions favorable for G4 stabilization [64,65]. G4↔Watson-Crick duplex equilibrium depends on many factors that change the relative stability of competing secondary structures. These include cationic composition, G4 topology, molecular crowding conditions (PEG 200 added to a standard quadruplex buffer promotes G4 formation), the presence of small-molecule G4-specific ligands, and the length of the hybridization site (G4 loops longer than 1-nt ones and the availability of fragments flanking a quadruplex structure (as in our G4 constructs) shift the structural equilibrium toward the DNA duplex formation) [32,66]. Only extremely stable parallel quadruplexes, such as *c-MYC* G4, with a limited loop length can form in long dsDNA under molecular crowding conditions and/or in the presence of G4-stabilizing small molecule ligands [67]. It has been shown that without molecular crowding, none of the G4-forming promoter sequences was able to sustain a stable G4 in a duplex context [68].

To estimate the secondary structure of hybridization products of 96-nt *hTERT* G4 constructs bearing various G>F substitutions with the complementary strands (C is opposite F), we performed CD measurements. The recorded CD spectra show features indicative of a G/C-rich DNA double helix (Supplementary Figure S3A) [69].

**Figure 5:**
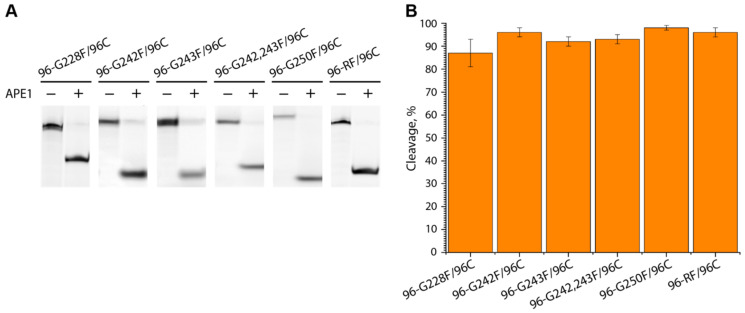
APE1-catalyzed F site cleavage in double-stranded 96-bp *hTERT* promoter constructs. Reaction mixtures consisted of 20 mM Tris-HCl (pH 7.5), 100 mM KCl, 5 mM MgCl_2_, 1 mM DTT, 0.5 mg/mL BSA, 100 nM 3′-TAMRA-labeled dsDNA substrate, and 10 U (36 nM) of APE1. The reactions were carried out at 37 °C for 60 min and quenched by heating at 60 °C for 20 min. (**A**) Representative gel images of 3′-TAMRA-labeled cleavage products. (**B**) Cleavage efficiency as a function of the lesion position as determined from the PAGE data. Error bars are 95% confidence intervals (n = 3).

Comparative data on the efficiency of the APE1-catalyzed reaction in ss and ds 96-mer *hTERT* G4s (Figure 4 and Figure 5A,B) indirectly confirmed the disappearance of the G4 structure after the conversion of *hTERT* G4 motifs into ds variants. Thus, the final yields of 96-G228F/96C, 96-G250F/96C, 96-G242F/96C, 96-G243F/96C, and 96-G242,243F/96C cleavage products were 85–100% and did not depend significantly on the F site’s position within the *hTERT* G4 motif (Figure 5A,B). Our results are entirely consistent with a recent report that APE1 processes AP sites in the folded *VEGF* G4 promoter much less efficiently as compared to the ds *VEGF* promoter lacking a quadruplex structure [15]. Hence, the formation and stability of G4 structure, rather than the sequence of the DNA substrate, largely affects the endonuclease activity of APE1.

### 2.5. Efficiency of APE1 Binding to the 96-nt hTERT G4 and Its G>F Substitution–Bearing Analogs

To test whether the poor APE1 endonuclease activity observed on F-containing *hTERT* G4 substrates results from compromised enzyme–substrate complex formation, we determined *K*_d_ for APE1 binding to various DNA substrates. The affinity of the enzyme for the AP site is approximately 70 times stronger than for undamaged DNA [70]. APE1 binds ssDNA or dsDNA of various lengths (~10 to 63 bp; the lower limit is the enzyme footprint size) in the presence of divalent metal cations [71]. Nevertheless, the stability of APE1–DNA complexes diminishes rapidly with increasing ionic strength [72].

**Figure 6:**
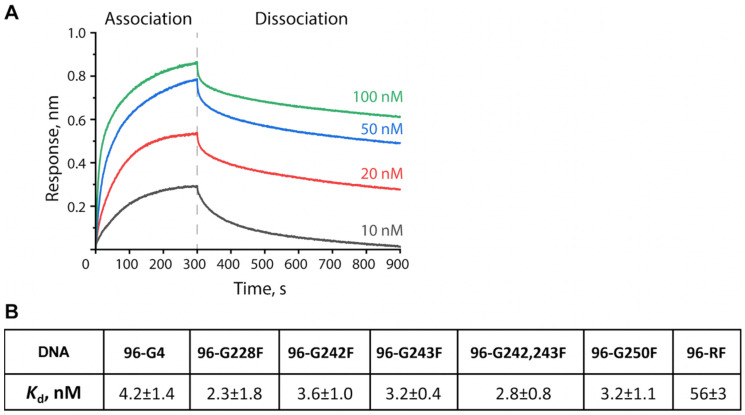
Binding of the APE1 enzyme to 96-nt *hTERT* G4 constructs containing F site(s). (**A**) Association and dissociation curves of the APE1–96-G242F complex as recorded by bio-layer interferometry. (**B**) *K*_d_ values of APE1 binding to 96-nt DNA substrates as determined by bio-layer interferometry.

We examined APE1–DNA interactions in real time by bio-layer interferometry (BLI) [73]. APE1 bearing a His-tag was immobilized on a Ni-NTA-coated biosensor surface. Binding assays were performed in the presence of Ca^2+^ instead of Mg^2+^ to avoid DNA cleavage [74]. Complex formation with 96-nt *hTERT* G4 constructs carrying single G>F substitutions was carried out for 300 s (association stage). The dissociation stage was implemented in the same buffer for 600 s (Figure 6A). APE1 manifested robust binding to 96-G228F, 96-G242F, 96-G243F, 96-G242,243F, and 96-G250F with similar *K*_d_ values (~3 nM), regardless of F site location. A slightly higher *K*_d_ value of 4.2 ± 1.4 nM was observed for the APE1 complex with unmodified 96-G4, whereas F-containing 96-RF, lacking a quadruplex structure, showed much weaker affinity, with its *K*_d_ value being an order of magnitude higher than that of the APE1 complexes with G4 substrates (Figure 6B).

### 2.6. Efficiency of APE1-Catalyzed Cleavage of the AP Sites in 30-nt hTERT ^2^G4 Constructs

To better understand how the AP site position in a single G4 affects the ability of APE1 to process the lesion, we next studied the APE1-induced cleavage of the 30-nt *hTERT* ^2^G4 (the central quadruplex unit) harboring all types of G>F substitutions (Supplementary Table S1) under the same conditions (100 mM KCl), as for 96-nt ssDNA (Supplementary Table S2).

**Figure 7:**
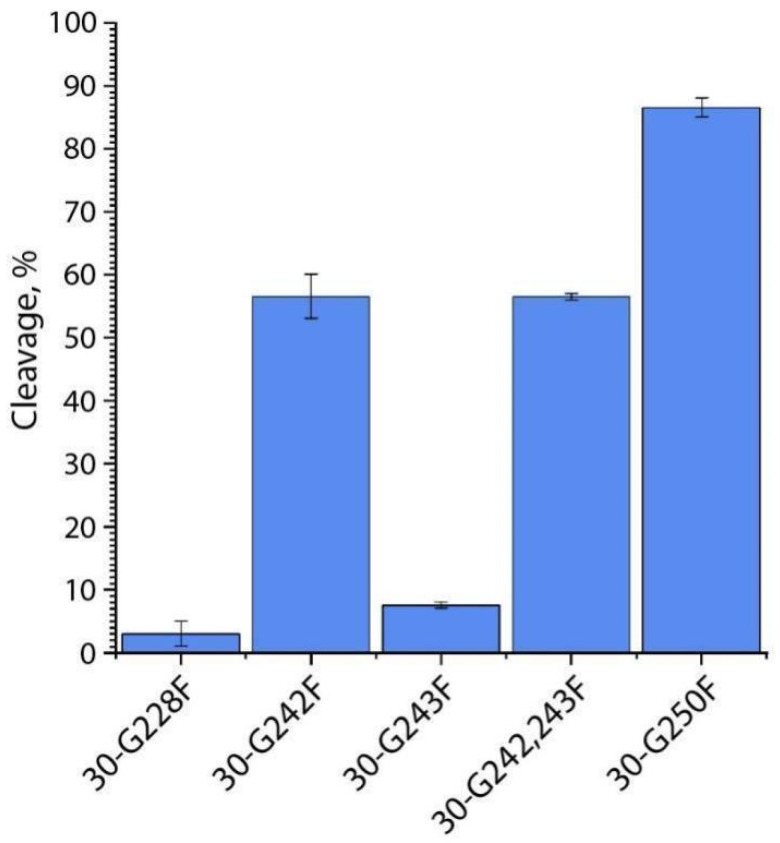
The APE1-induced cleavage of the AP (F) site in 30-nt *hTERT* ^2^G4 constructs. Reaction mixtures consisted of 20 mM Tris-HCl (pH 7.5), 100 mM KCl, 5 mM MgCl_2_, 1 mM DTT, 0.5 mg/mL BSA, 100 nM 3′-TAMRA-labeled ssDNA substrate, and 10 U (36 nM) of APE1. The reactions were allowed to proceed at 37 °C for 60 min and quenched by heating at 60 °C for 20 min. Error bars are 95% confidence intervals (n = 2).

Without the masking effect of flanking quadruplexes in the full-length 96-nt *hTERT* G4 constructs, the 30-nt quadruplex substrates manifested a greater impact of the abasic site on G4 destabilization and showed a clear inverse correlation between G4 stability and APE1-induced cleavage efficiency (compare Figure 3B and Figure 7; Spearman’s ρ = −0.973, *p* < 0.05). The yield of products varied from 3–7% for the most stable substrates, 30-G228F and 30-G243F (*T*_m_ of the quadruplex structure was 68 °C, which reflects a minimal destabilizing effect of the AP site located at terminal positions of the 5- and 4-nt G-tracts, respectively), to ~50% for 30-G242F and 30-G242,243F with a single or double substitution in the middle of the 4-nt G-tract (*T*_m_ = 63 °C), to 88% for the least stable 30-G250F (*T*_m_ = 58 °C). Thus, the yield of APE1-catalyzed F site cleavage in the *hTERT* ^2^G4 context is highly dependent on the location of the lesion in the quadruplex scaffold.

### 2.7. Reduced APE1-Catalyzed DNA Cleavage in the ds 96-bp hTERT Promoter Region Carrying AP (F) Sites in the C-Rich Strand

Our hypothesis postulates that the occurrence of AP sites in the G4 motifs of the *hTERT* promoter is one of the sources of the high mutation frequency in this genome region. Nevertheless, AP sites can also appear in the complementary C-rich strand of the *hTERT* promoter as a consequence of C deamination to U followed by excision by one of the human uracil DNA glycosylases [2]. Because AP sites in a C-rich strand strongly destabilize the double-helical structure and shift the structural equilibrium in favor of G4 folding [75], we expected that G4s would form in the G-rich strand, thereby effectively impeding the repair at the AP site processing stage.

The CD spectra of 96-bp double-stranded versions of the *hTERT* promoter region containing C>F substitutions at positions of the *hTERT* driver mutations (Supplementary Table S2) were found to correspond to the spectra of a mixture of G/C-rich DNA duplexes and G4 structures that were formed by the unhybridized G-rich strand of the *hTERT* promoter region (Supplementary Figure S3B).

**Figure 8:**
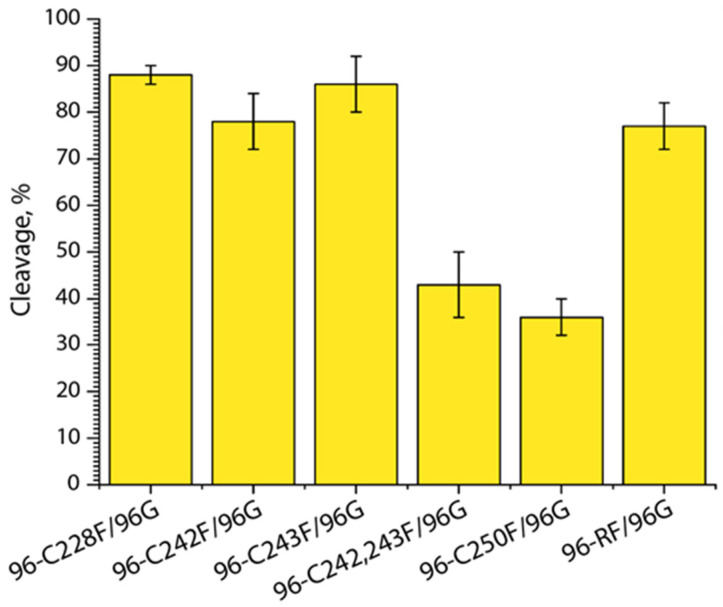
Cleavage of the C-rich strand bearing C>F substitutions in duplex DNA. Reaction mixtures consisted of 20 mM Tris-HCl (pH 7.5), 100 mM KCl, 5 mM MgCl_2_, 1 mM DTT, 0.5 mg/mL BSA, 100 nM 3′-TAMRA-labeled dsDNA substrate, and 10 U (36 nM) of APE1. The reactions were allowed to proceed at 37 °C for 60 min and quenched by heating at 60 °C for 20 min. Error bars are 95% confidence intervals (n = 2).

We next examined the efficiency of AP site processing by APE1 in these double-stranded constructs that model the *hTERT* promoter region. Reaction mixtures were composed of 20 mM Tris-HCl (pH 7.5), 100 mM KCl, 5 mM MgCl_2_, 1 mM DTT, 0.5 mg/mL BSA, 100 nM 3′-TAMRA-labeled DNA substrate, and 10 U (36 nM) of APE1. The reactions were carried out at 37 °C for 60 min and quenched by heating at 60 °C for 20 min. The yields of cleavage products of DNA duplexes with G>F substitutions reached 85–100% regardless of the F site location in the *hTERT* G4 motif (Figure 5A,B). Nevertheless, the efficiency of DNA cleavage in the C-rich strand bearing C>F substitutions depended on the position of the abasic site and approached 80–90% in the case of 96-C228F/96G, 96-C242F/96G, 96-C243F/96G, and 96-RF but remained at or below 40% for 96-C242,243F/96G and 96-C250F/96G (Figure 8).

## 3. Discussion

APE1 is the main repair protein for DNA abasic lesions in mammals. This enzyme catalyzes the cleavage of the phosphodiester backbone at the AP site—the first common intermediate in BER—thus playing a central role in the entire pathway [24,25,26]. This enzyme is also involved in transcription regulation, thereby modulating gene expression [16,17,76]. Recently, it was shown that the efficiency of APE1-induced cleavage of AP sites is influenced by the secondary structure of DNA, in particular by noncanonical G4 structures harboring this damaged site [77,78]. Here we analyzed the G/C-rich promoter region of the *hTERT* oncogene, whose G-rich strand carries 12 consecutive G-tracts that are capable of folding into three stacked parallel G4s in vitro [53,54,61]. The rationale for our study was the hypothesis that the high mutation frequency in G/C-rich promoters is explained by quadruplex-driven attenuation of the activity of a repair protein, in particular, APE1. Quantitative proteomic data suggest that human APE1 is a very abundant nuclear protein (by far the most abundant among BER proteins) [79], and therefore owing to its strong affinity for G4 DNA [15,31,47,80], this enzyme is likely to interact with many damaged G4s. Although the possibility of preferential C deamination as a source of mutations in the *hTERT* promoter was recently addressed [81], no data have been reported regarding APE1 activity on an AP site-carrying *hTERT* multiquadruplex.

After a bioinformatic analysis of the genetic materials of cancer patients from the IGCC database, we did not find a correlation between mutations in *hTERT* and *APEX1* (Table 1). This finding essentially rules out the possibility that the high genetic instability in the promoter region of the *hTERT* gene is due to APE1 dysfunction caused by amino acid substitutions. Consequently, the main focus of our investigation was on the activity of wild-type APE1 in G4 DNA structures.

To understand the effect of a G4 on APE1, we studied interactions of APE1 with 96-nt *hTERT* G4 constructs that form a three-quadruplex tandem or with a 30-nt *hTERT* G4 capable of folding into a central quadruplex. These model DNAs harbored G>F substitutions (where F is an AP site analog that is resistant to spontaneous decay) at positions matching major driver mutations (Supplementary Table S1, Figure 1B). As evident from CD spectroscopy data (Figure 2 and Figure 3), single G>F substitutions did not prevent quadruplex folding yet changed its topology and stability owing to local rearrangement of base pairing and stacking interactions in the G4 core. The presence of G-tracts longer than 3 nt is a potential source of quadruplex structural polymorphism. Although unmodified construct 96-G4 manifested characteristics of a typical G4 parallel fold, some G>F substitutions within the quadruplex scaffold promoted an antiparallel (hybrid) G4 topology. Nonetheless, the parallel directionality of the G-tracts predominated even in a quadruplex structure harboring an AP site. The destabilizing effect was strongly dependent on the G-tracts’ length and the lesion’s position within the tract; the destabilization was less pronounced if the AP site could be excluded from the G4 core by means of redundant G residues in a long (4- or 5-nt) G-tract. The greatest impact on G4 destabilization was exerted by the G>F substitution at position 250, located in the 3-nt G-tract, and by the double G>F substitution at positions 242 and 243 (Figure 2). We have previously researched the effect of G>A substitutions located at the same positions of driver mutations in 96-nt *hTERT* G4 constructs. Those observations [55] are in agreement with the present results: Significant destabilization of G4 occurred after substitution G250A, located in the 3-nt G-tract. More pronounced positional dependence of AP site–induced G4 destabilization was observed with the 30-nt *hTERT* G4 constructs corresponding to the central ^2^G4, apparently because the absence of flanking G4 units made the destabilization effect more obvious (Figure 2).

Monitoring of APE1-catalyzed AP site cleavage revealed the following trend for both 96- and 30-nt *hTERT* G4 constructs: enzymatic activity increased when the thermal stability of the F-containing *hTERT* G4 structure decreased (Figure 4 and Figure 7). A clear inverse correlation between G4 stability and APE1-induced cleavage efficiency was documented for 30-nt quadruplex substrates (compare Figure 3B and Figure 7); the yield of DNA cleavage products varied from 3–7% for the stablest 30-G228F and 30-G243F to 88% for the least stable 30-G250F. As expected, all DNA duplex substrates were robustly cleaved when a complementary strand was added to the 96-nt quadruplex-forming DNA molecules to prevent G4 folding (Figure 5A,B). CD data confirmed that 96-bp double-stranded versions of *hTERT* promoter constructs with various G>F substitutions give rise predominantly to a G/C-rich DNA duplex (Supplementary Figure S3A) [69]. A major limitation of the in vitro assay used in this study is the absence of numerous cellular factors that may influence APE1 functions. Thus, in vitro studies, which cannot fully mimic the complexity of cellular processes, represent only a first step in understanding the molecular mechanisms of *hTERT*-driven mutation maintenance. On the other hand, the in vitro approach allowed us to quantitatively study the position-dependent effect of AP sites in the quadruplex structure on APE1 activity.

APE1 binds in the minor groove of an abasic site (F)-containing DNA duplex and kinks the double helix axis, thereby covering the 5′-NFNNN-3′ stretch on the damaged strand and entering into even more extensive contacts with nucleotides in the complementary strand [19,20]. Therefore, it is very likely that G4 DNA cannot be easily switched by the enzyme to a catalytically competent conformation. When the quadruplex is partially unfolded, the AP site may end up in an unpaired single strand and be cleaved by APE1, albeit with lower efficiency than that in duplex DNA [18,27,28]. Accordingly, the endonuclease activity of APE1 is largely influenced by the formation and stability of the G4 structure rather than by the sequence of the DNA substrate.

Despite poor processing of AP site-carrying 96-nt *hTERT* G4s, the APE1 enzyme showed tight binding to these quadruplex substrates with similar *K*_d_ values (~3 nM) regardless of the F site presence and location, as determined by bio-layer interferometry. At the same time, F-containing 96-RF lacking a quadruplex structure was bound by APE1 with much lower efficiency; *K*_d_ was an order of magnitude higher than that of the G4 folded substrates (Figure 6B). Overall, our data are consistent with the literature (mostly about the *VEGF* promoter and telomeric G4s) indicating that APE1 has strong affinity for DNA G4s even in the absence of AP sites; this phenomenon may be important for (i) understanding the role of a G4 in reactivation of transcription factors in promoter regions [15,31,47,80] and (ii) assessing the influence of promoter quadruplexes on gene expression.

Our findings suggest that G4 structures suppress AP site repair, regardless of whether the lesion is in the G- or C-rich strand of the *hTERT* promoter region (Figure 8). In the former case, the quadruplex structure sterically hinders the formation of a productive enzyme–substrate complex. Even though AP sites destabilize the G4, this is unlikely to be enough to drive DNA back to the duplex structure, which is the best APE1 substrate. In the latter case, AP sites in the C-rich strand significantly destabilize the duplex, thereby shifting the structural equilibrium in favor of G4 formation (Supplementary Figure S3B) [75]. In both cases, fairly stable G4 structures tightly bind the APE1 enzyme and prevent it from efficient repair of AP sites.

**Scheme 1:**
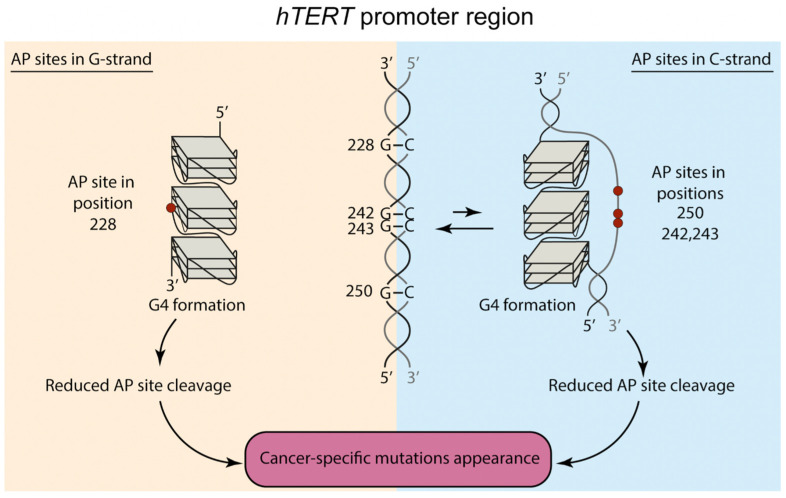
Schematic representation of our findings that G4 structures suppress AP site repair, regardless of whether the lesion is in the G- or C-rich strand of the *hTERT* promoter region.

Our findings support the notion that APE1 can bind favorably to G4 structures, regardless of whether they contain an AP site, but G4 recognition and robust binding are not related to lesion correction, thus leading to the accumulation of genomic mutations (Scheme 1). In the future, we plan to support our hypothesis by studying the crosstalk between *hTERT* G4 and 8-oxoguanine-DNA glycosylase-mediated repair of oxidative base lesions located at various positions of the quadruplex core.

## 4. Materials and Methods

### 4.1. Bioinformatic Analysis of Somatic Mutations in Isolates of Cancer Patients

To investigate whether mutations in the *APE1* gene and *hTERT* promoter occur simultaneously, we analyzed simple somatic mutation (SSM) files from the 28th DCC Data Release of the International Cancer Genome Consortium (ICGC) database, using the following cancer projects: BOCA-FR (Soft Tissue cancer—Ewing sarcoma—France), BOCA-UK (Bone Cancer—UK), BTCA-SG (Biliary Tract Cancer—SG), CLLE-ES (Chronic Lymphocytic Leukemia—ES), LICA-CN (Liver Cancer—CN), LICA-FR (Liver Cancer—FR), LIHM-FR (Liver Cancer—Hepatocellular macronodules—FR), LINC-JP (Liver Cancer—JP), MELA-AU (Skin Cancer—AU), ORCA-IN (Oral Cancer—IN), PBCA-DE (Pediatric Brain Cancer—DE), PBCA-US (Pediatric Brain Tumor—US), PEME-CA (Pediatric Medulloblastoma—CA), RECA-EU (Renal Cell Cancer—EU), and SKCA-BR (Skin Adenocarcinoma—BR). Thus, we were able to search for the presence of mutations in the *hTERT* promoter and the *APEX1* gene encoding APE1, located on chromosomes 5 and 14, respectively, in the genome of each given patient by matching the donor ID in the mutation data files. The custom script used for this analysis can be found at this link: https://colab.research.google.com/drive/1WHPrLjaXjOaodrdLnj4PZ2kFvUkYFFOH?usp=sharing, accessed on 24 November 2024]. In our work, we utilized the ratio of the number of donors carrying mutations in both *hTERT* and *APEX1* genes to the number of all donors in the cancer project. This type of calculation does not take into account the number of *APEX1* mutations (which varies from donor to donor) but reflects the existence of simultaneous mutations in the donor. SKCA-BR was chosen as the control group for the Fisher exact test because this group was comparable in size to MELA-AU and did not contain simultaneous mutations in the script results.

### 4.2. DNA Sample Preparation

All oligodeoxyribonucleotides (Supplementary Table S1), including those labeled with tetramethylrhodamine (TAMRA), were synthesized and purified at Syntol (Moscow, Russia). The concentration of oligonucleotides was determined spectrophotometrically on a Hitachi U-2900 double-beam UV-visible spectrophotometer (Hitachi, Japan). Molar extinction coefficients were derived from nearest-neighbor data (https://www.idtdna.com/calc/analyzer, accessed on 10 December 2022).

To generate intramolecular *hTERT* G4s with or without G>F substitutions, 30- or 96-nt 3′-TAMRA-labeled ssDNA samples were annealed in a buffer containing at least 20 mM KCl by heating at 98 °C for 20 min and were slowly cooled to 4 °C on a heat block. DNA duplexes were prepared by the addition of complementary DNA strands to G4-forming oligonucleotides; the resulting mixtures were annealed via heating to 95 °C for 5 min and then were slowly cooled to room temperature. The unlabeled DNA strand was used in a 10% excess over the TAMRA-labeled strand.

### 4.3. CD Measurements

Next, 96-nt ssDNA samples containing the WT *hTERT* G4 motif or G>F substitution-containing versions (Supplementary Table S1) were annealed in 8-mM potassium phosphate buffer (pH 7.1) with 20 mM KCl. For the corresponding 30-nt DNAs (Supplementary Table S1) that are capable of forming a central G4 unit, the annealing procedure was performed in 10-mM Tris-HCl buffer (pH 8.0) with 1-mM EDTA and 100 mM KCl. CD experiments were conducted using a 1-cm optical-path length quartz cuvette at 30 °C on a Chirascan CD spectrometer (Applied Photophysics Ltd., Leatherhead, UK) equipped with a Peltier temperature controller. Quadruplex melting temperature (*T*_m_) was determined from the CD-monitoring-derived thermal denaturation profiles of the 96-nt and 30-nt *hTERT* G4 structures at 265 nm. CD spectra were recorded between 30 and 85 °C with a temperature interval of ~5 °C at an average heating rate of 0.5 °C/min. DNA concentrations were typically 2 µM per strand. Data were collected in the wavelength range 230–360 nm at a scan rate of 30 nm/min and a bandwidth of 0.5 nm under a constant stream of dry nitrogen. The spectrum of the buffer was subtracted from each sample’s spectrum. CD spectra were plotted as molar dichroism per oligonucleotide strand (or DNA duplex) as a function of wavelength. Data processing was carried out in Origin 8.0 software (Electronic Arts, Redwood City, CA, USA). CD melting profiles were generated via plotting of temperature-dependent CD signals at 265 nm.

### 4.4. APE1 Enzyme Isolation

The human APE1 enzyme was isolated from *Escherichia coli* Rosetta 2 cells transformed with the pET11a plasmid carrying the human AP endonuclease gene. Human APE1 was purified essentially as described before [61]. The enzyme concentration was calculated from the absorbance of the protein at 280 nm and the molar extinction coefficient of 56,818 M^–1^cm^–1^.

### 4.5. DNA Cleavage by APE1

Reaction mixtures (10 µL) consisted of 20 mM Tris-HCl (pH 7.5), 100 mM KCl, 5 mM MgCl_2_, 1 mM DTT, 0.5 mg/mL BSA, 100 nM 3′-TAMRA-labeled DNA substrates (96- and 30-nt ssDNA samples or 96- and 30-bp DNA duplexes; the TAMRA fluorophore is located in one of the strands) and 10 U (36 nM) APE1. One unit of the enzyme was defined as the amount of APE1 that cleaves 20 pmol of a DNA duplex carrying a single AP site (10 µL, 1 h, 37 °C). Reactions were carried out at 37 °C for 60 min and quenched by heating at 60 °C for 20 min. The reaction products were analyzed by electrophoresis in a denaturing 20% polyacrylamide gel. TotalLab TL120 Image software, version 2.01 (Nonlinear Dynamics Ltd., New Castle, UK) was used to visualize DNA cleavage products.

### 4.6. DNA-Binding Activity of APE1

The ability of APE1 to bind TAMRA-labeled DNA probes was studied by biolayer interferometry by means of a BLItz system (ForteBio, Fremont, CA, USA) according to the manufacturer’s protocol. Briefly, His-tagged APE1 was coupled to a Ni-NTA biosensor via soaking of the sensor in 3.5 µM APE1 for 5 min. The association step of DNA probes (10, 20, 50, and 100 nM) was analyzed for 5 min in a buffer composed of 20 mM Tris-HCl (pH 7.5), 100 mM KCl, and 1 mM CaCl_2_ with constant stirring at 1000 rpm. The data were processed in BLItz Pro v1.3.0.5 software.

Dissociation constants (*K_d_*) of the complexes were calculated using the following equations:

(1) $$R=R_{eq}\left(1-e^{-\left(k_{a}\cdot C+k_{d}\right)\left(t-t_{0}\right)}\right)$$

which is the integral equation for the association rate;

(2) $$R=R_{0}\cdot e^{-k_{d}\left(t-t_{0}\right)}+R_{\left(t\to \infty \right)}$$

which is the integral equation for the dissociation rate;

(3) $$K_{d}=\frac{k_{d}}{k_{a}}$$

where *R* is the spectrometer response (wavelength shift, nm) at any time point, *R_eq_* is the spectrometer response corresponding to the equilibrium stage, *R*_0_ is the spectrometer response representing the initial stage of dissociation, *R*_(*t*→∞)_ is the spectrometer response for infinite time, *C* is the analyte concentration, *k_a_* and *k_d_* are association and dissociation rate constants, respectively, and *t* is time (s).

## 5. Conclusions

Our results add the human *hTERT* promoter to the growing list of gene expression-regulatory elements harboring potential G4-forming sequences that respond to DNA damage by conformational changes and recruit APE1, likely as part of transcription initiation control machinery. We propose the following mechanism. AP sites in any *hTERT* promoter strand destabilize the DNA duplex and promote G4 formation. If the lesion is in a G-rich strand, then the quadruplex structure binds APE1 but is poorly repaired, allowing APE1 to be sequestered on G4. If the lesion is in a C-rich strand, then the quadruplex binds APE1, but the AP site in the exposed C-rich strand persists. In any case, prolonged damage is at a high risk of being converted into a driver mutation that activates *hTERT* in precancerous cells. Nonetheless, this repair deficiency varies depending on several factors, including the DNA duplex strand in which the damage occurs, the number of G residues in the G-tract, and the presence of nearby G-tracts. The efficiency of AP site removal from G4s by APE1 ultimately depends on the stability of the G4 structure. According to our bioinformatic analysis, the high mutation frequency in the *hTERT* promoter is not a consequence of APE1 dysfunction caused by amino acid substitutions in the enzyme active site.

## Figures and Tables

**Table 1:** Percentages of patients with simultaneous mutations in the *hTERT* promoter and APEX1 gene.

Position of *hTERT* Promoter Mutation	Patients with Simultaneous Mutations in the *hTERT* and *APEX1*
G228	1 out of 122 (0.81%)
G242	1 out of 122 (0.81%)
G243	1 out of 122 (0.81%)
G250	2 out of 122 (1.63%)

## Data Availability

Data associated with this manuscript are available in the article and Supplementary Materials. Please contact the corresponding author to access the additional unpublished data.

## Supplementary Materials

The following supporting information can be downloaded at: https://www.mdpi.com/article/10.3390/ijms26010337/s1.
