# Supplementary material for: Position-Dependent Effects of AP Sites Within an hTERT Promoter G-Quadruplex Scaffold on Quadruplex Stability and Repair Activity of the APE1 Enzyme
Source: Int J Mol Sci. 2025 Jan 2;26(1):337. doi: 10.3390/ijms26010337 (PMC11720163; doi:10.3390/ijms26010337)
Supplement: Supplementary file 1 [file ijms-26-00337-s001.zip › ijms-3361766-supplementary.pdf]

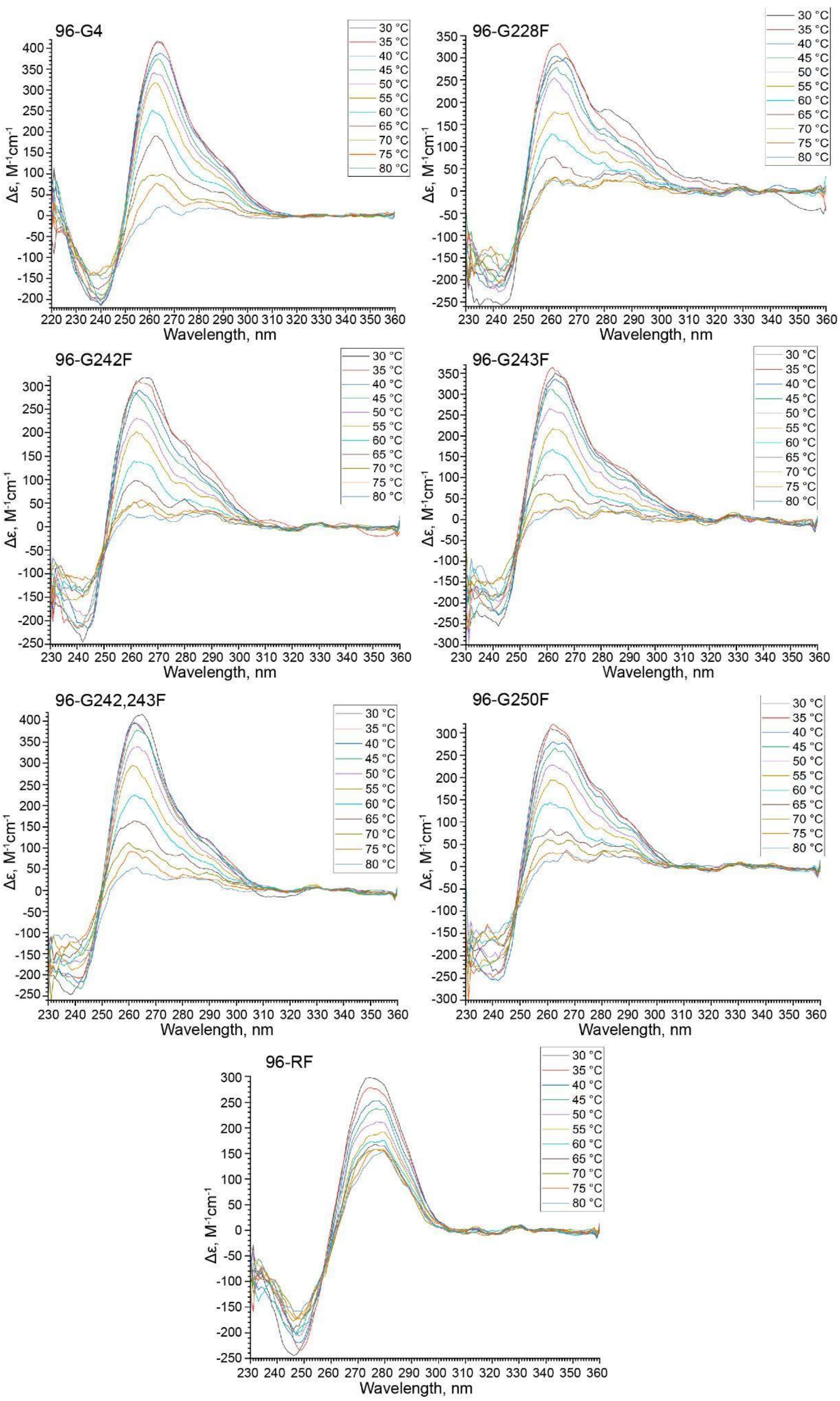

Supplementary Figure S1. CD spectra of 96-nt G-rich *hTERT* promoter constructs that differ in the position of the G>F substitutions were recorded at different temperatures in 8 mM K-phosphate buffer (pH 7.1) containing 20 mM KCl. The temperature was increased from 30 to 80 °C in 5 °C steps; colored lines show the CD spectra at different temperatures.

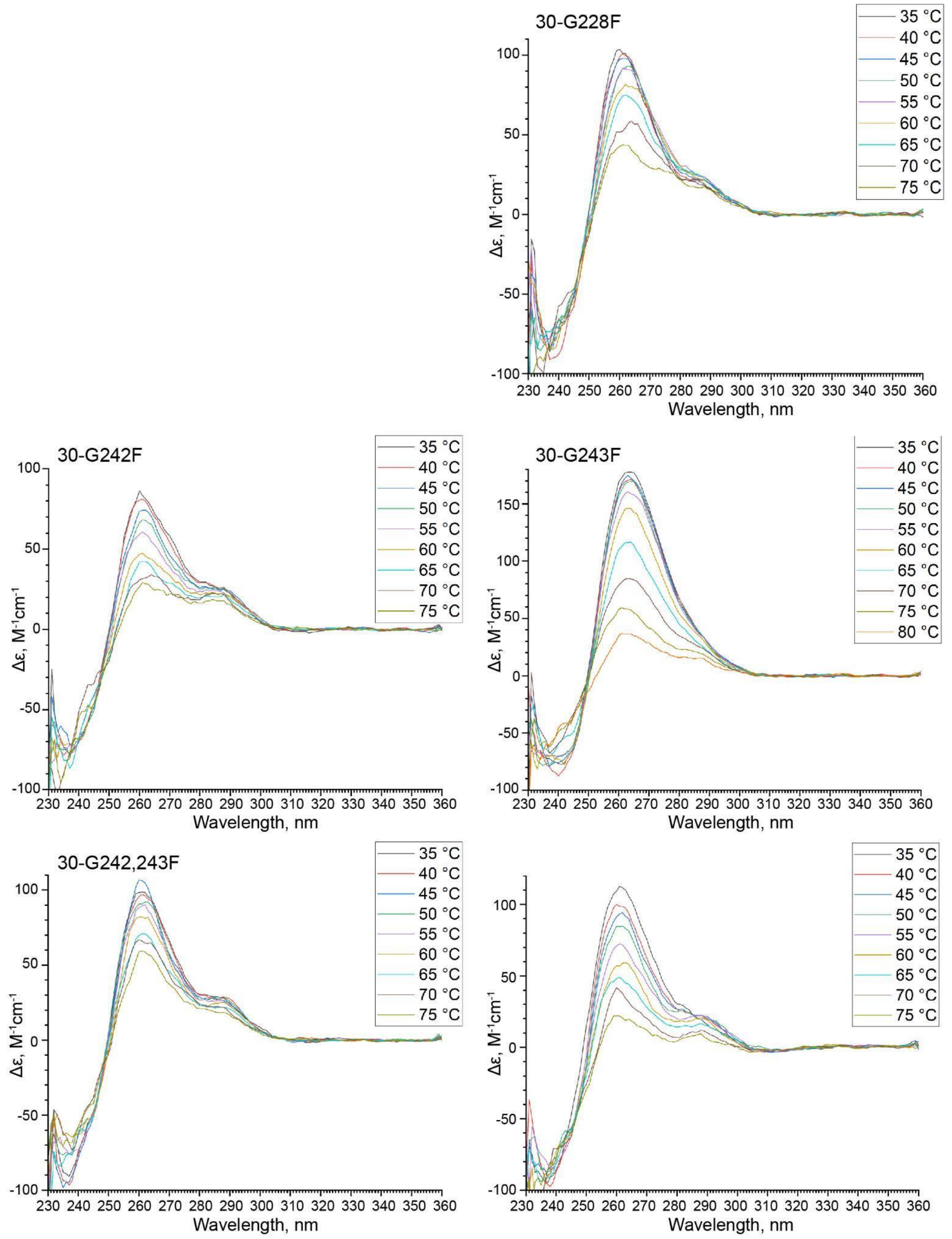

Supplementary Figure S2. CD spectra of 30-nt G-rich *hTERT* promoter constructs bearing G>F substitutions were recorded at different temperatures in 10 mM Tris-HCl buffer (pH 8.0) containing 1 mM EDTA and 100 mM KCl. This is the buffer used to analyze the interaction of APE1 with *hTERT* G4. Temperature increased from 30 to 85 °C in 5 °C steps; multicolor lines show the CD spectra at different temperatures.

A)

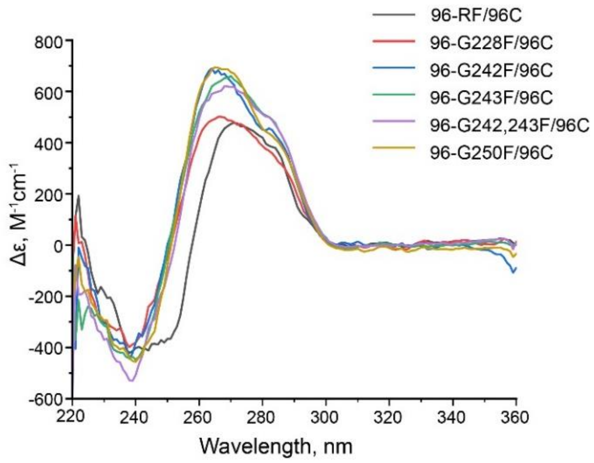

B)

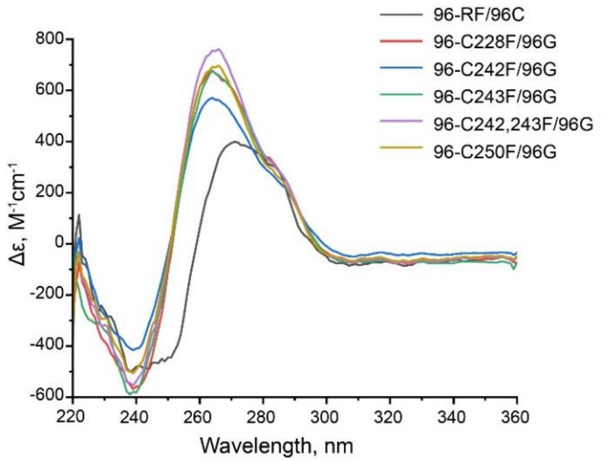

Supplementary Figure S3. CD spectra of double-stranded versions of the 96-bp G/C-rich *hTERT* promoter region that differ in the position of the AP (F) site were recorded at 30 °C in 8 mM K-phosphate buffer (pH 7.1) containing 20 mM KCl. (**A**) 96-bp *hTERT* promoter dsDNA bearing G>F substitutions (C is opposite F). (**B**) 96-bp *hTERT* promoter dsDNA bearing C>F substitutions (G is opposite F).

Supplementary Table S1. Abbreviation and sequence of the synthetic the TAMRA-labeled DNA oligonucleotides used in this study. Modification (F) highlighted in red.

Oligonucleotide	Sequence
96-G4	5′- GCCGCGGAAAGGAAGGGGAGGGGCTGGAGGGCCCGGAGGGGGCTGGGCCGGGGACCCGGGAGGGGTCGGGACGGGGCGGGGTCCGCGCGGAGGAG-3′-TAMRA
96-RF	5′- ACCTGGATGCCTATAGGGCGAATTGGGTACCGCTGAATTGCACTGGACT**F**GATCCTCGATGATCCTAAGCTAAGCTTCAGCTCCAGCCTAAGCCTG-3′-TAMRA
96-G228F	5′- GCCGCGGAAAGGAAGGGGAGGGGCTGGAGGGCCCGGA**F**GGGGCTGGGCCGGGGACCCGGGAGGGGTCGGGACGGGGCGGGGTCCGCGCGGAGGAG-3′-TAMRA
96-G242F	5′- GCCGCGGAAAGGAAGGGGAGGGGCTGGAGGGCCCGGAGGGGGCTGGGCCG**F**GGACCCGGGAGGGGTCGGGACGGGGCGGGGTCCGCGCGGAGGAG-3′-TAMRA
96-G243F	5′- GCCGCGGAAAGGAAGGGGAGGGGCTGGAGGGCCCGGAGGGGGCTGGGCCGG**F**GACCCGGGAGGGGTCGGGACGGGGCGGGGTCCGCGCGGAGGAG-3′-TAMRA
96-G242,243F	5′- GCCGCGGAAAGGAAGGGGAGGGGCTGGAGGGCCCGGAGGGGGCTGGGCCG**FF**GACCCGGGAGGGGTCGGGACGGGGCGGGGTCCGCGCGGAGGAG-3′-TAMRA
96-G250F	5′- GCCGCGGAAAGGAAGGGGAGGGGCTGGAGGGCCCGGAGGGGGCTGGGCCGGGGACCCGG**F**AGGGGTCGGGACGGGGCGGGGTCCGCGCGGAGGAG-3′-TAMRA
30-RF	5′- TGAATTGCACTGGACT**F**GATCCTCGATGAT-3′-TAMRA
30-G228F	5′- CCGGA**F**GGGGCTGGGCCGGGGACCCGGGAG-3′-TAMRA
30-G242F	5′- CCGGAGGGGGCTGGGCCGG**F**GACCCGGGAG-3′-TAMRA
30-G243F	5′- CCGGAGGGGGCTGGGCCGGG**F**ACCCGGGAG-3′-TAMRA
30-G242,243F	5′- CCGGAGGGGGCTGGGCCGG**FF**ACCCGGGAG-3′-TAMRA
30-G250F	5′- CCGGAGGGGGCTGGGCCGGGGACCCGG**F**AG-3′-TAMRA
41-RF	5′- CTGAGCCATATTTGTGTGTGT**F**TGTGTGTTGCTCGAGTAAC-3′-TAMRA

Supplementary Table S2. Abbreviation and sequence of the synthetic DNA duplexes (left and middle columns) formed by hybridization of oligonucleotides (right) oligonucleotides used in this study. Modification (F) highlighted in red.

DNA duplex	Sequences	Oligonucleotide
96-RF/96C	5′-ACCTGGATGCCTATAGGGCGAATTGGGTACCGCTGAATTGCACTGGACT**F**GATCCTCGATGATCCTAAGCTAAGCTTCAGCTCCAGCCTAAGCCTG-3′ -TAMRA<br>3′-TGGACCTACGGATATCCCGCTTAACCCATGGCGACTTAACGTGACCTGACCTAGGAGCTACTAGGATTCGATTCGAAGTCGAGGTCGGATTCGGAC-5′	96-RF<br>96-C
96-G228F/96C	5′-GCCGCGGAAAGGAAGGGGAGGGGCTGGAGGGCCCGGA**F**GGGGCTGGGCCGGGGACCCGGGAGGGGTCGGGACGGGGCGGGGTCCGCGCGGAGGAG-3′-TAMRA<br>3′-CGGCGCCTTTCCTTCCCCTCCCCGACCCTCCCGGGCCTCCCCCGACCCGGCCCCTGGGCCCTCCCCAGCCCTGCCCCGCCCCAGGCGCGCCTCCTC-5′	96-228F<br>96-C
96-G242F/96C	5′-GCCGCGGAAAGGAAGGGGAGGGGCTGGAGGGCCCGGAGGGGGCTGGGCCGG**F**GACCCGGGAGGGGTCGGGACGGGGCGGGGTCCGCGCGGAGGAG-3′-TAMRA<br>3′-CGGCGCCTTTCCTTCCCCTCCCCGACCCTCCCGGGCCTCCCCCGACCCGGCCCCTGGGCCCTCCCCAGCCCTGCCCCGCCCCAGGCGCGCCTCCTC-5′	96-242F<br>96-C
96-G243F/96C	5′-GCCGCGGAAAGGAAGGGGAGGGGCTGGAGGGCCCGGAGGGGGCTGGGCCGGG**F**ACCCGGGAGGGGTCGGGACGGGGCGGGGTCCGCGCGGAGGAG-3′-TAMRA<br>3′-CGGCGCCTTTCCTTCCCCTCCCCGACCCTCCCGGGCCTCCCCCGACCCGGCCCCTGGGCCCTCCCCAGCCCTGCCCCGCCCCAGGCGCGCCTCCTC-5′	96-243F<br>96-C
96-G242,243F/96C	5′-GCCGCGGAAAGGAAGGGGAGGGGCTGGAGGGCCCGGAGGGGGCTGGGCCGG**FF**ACCCGGGAGGGGTCGGGACGGGGCGGGGTCCGCGCGGAGGAG-3′-TAMRA<br>3′-CGGCGCCTTTCCTTCCCCTCCCCGACCCTCCCGGGCCTCCCCCGACCCGGCCCCTGGGCCCTCCCCAGCCCTGCCCCGCCCCAGGCGCGCCTCCTC-5′	96-242,243F<br>96-C
96-G250F/96C	5′-GCCGCGGAAAGGAAGGGGAGGGGCTGGAGGGCCCGGAGGGGGCTGGGCCGGGGACCCGG**F**AGGGGTCGGGACGGGGCGGGGTCCGCGCGGAGGAG-3′-TAMRA<br>3′-CGGCGCCTTTCCTTCCCCTCCCCGACCCTCCCGGGCCTCCCCCGACCCGGCCCCTGGGCCCTCCCCAGCCCTGCCCCGCCCCAGGCGCGCCTCCTC-5′	96-250F<br>96-C
96-C228F/96G	5′-CTCCTCCGCGCGGACCCCGCCCCGTCCCGACCCCTCCCGGGTCCCCGGCCCAGCCCC**F**TCCGGGCCCTCCCAGCCCCTCCCCTTCCTTTCCGCGGC-3′-TAMRA<br>3′-GAGGAGGCGCGCCTGGGGCGGGGCAGGGCTGGGGAGGGCCCAGGGGCCGGGTCGGGGAGGCCCGGGAGGGTCGGGGAGGGGAAGGAAAGGCGCCG-5′	96-C228F<br>96-G
96-C242F/96G	5′-CTCCTCCGCGCGGACCCCGCCCCGTCCCGACCCCTCCCGGGTC**F**CCGGCCCAGCCCCCTCCGGGCCCTCCCAGCCCCTCCCCTTCCTTTCCGCGGC-3′-TAMRA<br>3′-GAGGAGGCGCGCCTGGGGCGGGGCAGGGCTGGGGAGGGCCCAGGGGCCGGGTCGGGGAGGCCCGGGAGGGTCGGGGAGGGGAAGGAAAGGCGCCG-5′	96-C242F<br>96-G
96-C243F/96G	5′-CTCCTCCGCGCGGACCCCGCCCCGTCCCGACCCCTCCCGGGT**F**CCCGGCCCAGCCCCCTCCGGGCCCTCCCAGCCCCTCCCCTTCCTTTCCGCGGC-3′-TAMRA<br>3′-GAGGAGGCGCGCCTGGGGCGGGGCAGGGCTGGGGAGGGCCCAGGGGCCGGGTCGGGGAGGCCCGGGAGGGTCGGGGAGGGGAAGGAAAGGCGCCG-5′	96-C243F<br>96-G
96-C242,243F/96G	5′-CTCCTCCGCGCGGACCCCGCCCCGTCCCGACCCCTCCCGGGT**FF**CCGGCCCAGCCCCCTCCGGGCCCTCCCAGCCCCTCCCCTTCCTTTCCGCGGC-3′-TAMRA<br>3′-GAGGAGGCGCGCCTGGGGCGGGGCAGGGCTGGGGAGGGCCCAGGGGCCGGGTCGGGGAGGCCCGGGAGGGTCGGGGAGGGGAAGGAAAGGCGCCG-5′	96-C242,243F<br>96-G
96-C250F/96G	5′-CTCCTCCGCGCGGACCCCGCCCCGTCCCGACCCCT**F**CCGGGTCCCCGGCCCAGCCCCCTCCGGGCCCTCCCAGCCCCTCCCCTTCCTTTCCGCGGC-3′-TAMRA<br>3′-GAGGAGGCGCGCCTGGGGCGGGGCAGGGCTGGGGAGGGCCCAGGGGCCGGGTCGGGGAGGCCCGGGAGGGTCGGGGAGGGGAAGGAAAGGCGCCG-5′	96-C250F<br>96-G
